# Cancer prevalence in Pakistan: meta-analysis of various published studies to determine variation in cancer figures resulting from marked population heterogeneity in different parts of the country

**DOI:** 10.1186/s12957-018-1429-z

**Published:** 2018-07-05

**Authors:** Romana Idrees, Saira Fatima, Jamshid Abdul-Ghafar, Ahmad Raheem, Zubair Ahmad

**Affiliations:** 1 Aga Khan University Hospital (AKUH), Karachi, Pakistan; 2 French Medical Institute for Mothers and Children (FMIC), Kabul, Afghanistan

**Keywords:** Cancer prevalence, Pakistan, Population heterogeneity, Cancer registry, Cancer incidence, Colorectal cancer, Gastric cancer, Oral cancer, Prostate cancer, Breast cancer

## Abstract

**Background:**

Pakistan’s population is ethnically diverse with distinct ethnic groups inhabiting various parts of the country. Cancer statistics obtained from specific regions populated by distinct ethnic groups may vary considerably. There is no national cancer registry. To determine whether there are indeed significant statistical differences in cancer incidence and prevalence, data was recorded from different parts of Pakistan based on the ethnic composition of the population in those parts.

**Methods:**

Ten papers (original articles) on cancer incidence and prevalence in Pakistan published in the last two decades were selected from PubMed and Google Scholar. Meta-analysis of findings of these studies was performed using Meta-analysis of Observational Studies in Epidemiology (MOOSE) checklist. χ^2^-based *I*^2^ test was used for evaluating heterogeneity and Forest plots were generated for calculating unadjusted prevalence estimates. Oral, gastric, prostate, breast, and colorectal cancers were selected for meta-analysis. *I*^2^ values of 75% or greater indicated high heterogeneity.

**Results:**

All five types of cancer selected for meta-analysis (performed on studies carrying similar statistical weights) showed extremely high heterogeneity with *I*^2^ values of 99.7% for oral cancer, 98.6% for prostate cancer, 98.3% for gastric cancer, 99.8% for breast cancer, and 85.4% for colorectal cancer. *p* values for all cancers were highly statistically significant.

**Conclusions:**

Our findings show that the prevalence rates of different cancer types demonstrate marked variation in different studies depending on the place of origin of the study and dominant ethnic group in that region, and these variations are highly statistically significant. A national cancer registry needs to be established as soon as possible.

## Background

Pakistan is a densely populated country located in South Asia with an estimated population of 190 million. Pakistan’s population is very ethnically diverse and heterogeneous and distinct ethnic groups inhabit specific regions in the country. At least six large ethnic groups constitute the bulk of the population. Owing to their heterogeneity, cancer statistics obtained from specific regions populated by distinct ethnic groups often vary considerably. The absence of a national cancer registry complicates the situation further. Thus, there are no national figures or data on incidence or prevalence of different cancers in the country. Various studies which have been published on cancer incidence or prevalence in Pakistan provide regional data that is often variable due to the peculiar ethnic composition of that specific region or area. In this study, we have analyzed and compared data from various studies published from different regions of Pakistan, which documented the commonest cancers in those particular areas with their particular ethnic makeup. The aim was to determine whether there are significant statistical differences in the data regarding cancer incidence and prevalence in different parts of Pakistan reflecting differences in the ethnic composition of the population in those parts especially in the absence of a national cancer registry.

## Methods

We searched for papers on cancer incidence and prevalence in Pakistan published in the last 20 years using databases including PubMed and Google Scholar. In addition, we used PAKmed to search for papers published in non-indexed local journals. Only papers published in English were included in the study. We applied different keywords as filters in our search (such as Pakistan, Cancer, Cancer registry, Cancer Incidence, Colorectal Cancer, Gastric Cancer, Oral Cancer, Prostate Cancer, Breast Cancer, Cancer epidemiology). A total of 13 original articles were selected for inclusion in the study. Review articles or case reports were not included. All 13 papers presented statistics on cancer from different regions of Pakistan with ethnically diverse and heterogeneous populations. We used the Meta-analysis of Observational Studies in Epidemiology (MOOSE) checklist for reporting the meta-analysis of 13 observational studies, which were selected for inclusion in the present study. The χ^2^-based *I*^2^ (*I* squared) test was used for evaluating the meta-analysis for heterogeneity. The *I*^2^ values used were 0% (no heterogeneity), 25% (low heterogeneity), 50% (moderate heterogeneity), and 75% (high heterogeneity). These were based on an article published in 2003. [13] We generated Forest plots for unadjusted prevalence estimates with 95% confidence intervals. *p* value of less than 0.05 was considered statistically significant. Oral (commonest cancer overall in males and females combined), gastric (relatively low incidence in spite of widespread helicobacter infection), colorectal (among the top five commonest cancers in males and females combined), breast (commonest cancer in females), and prostate (relatively low incidence in contrast to very high incidence worldwide) cancer were randomly selected for meta-analysis. STATA-12 software was used to analyze the data for pooled prevalence and to generate the Forest plots.

## Results

Full texts of 13 published papers on cancer incidence and prevalence in Pakistan were available for analysis. Thus, 13 studies were evaluated to describe the prevalence of cancer in Pakistan. The data were from five medium to large cities and divisions of the country including the two largest cities (five from Karachi and four from Lahore), and one study each from Hyderabad, Larkana, Multan, and Hazara divisions. The total number of cancer cases in these studies range from as low as 555 to as high as 80,390. The cancers selected for determining prevalence and heterogeneity were oral, prostate, gastric, breast, and colorectal cancer.

**Fig. 1:**
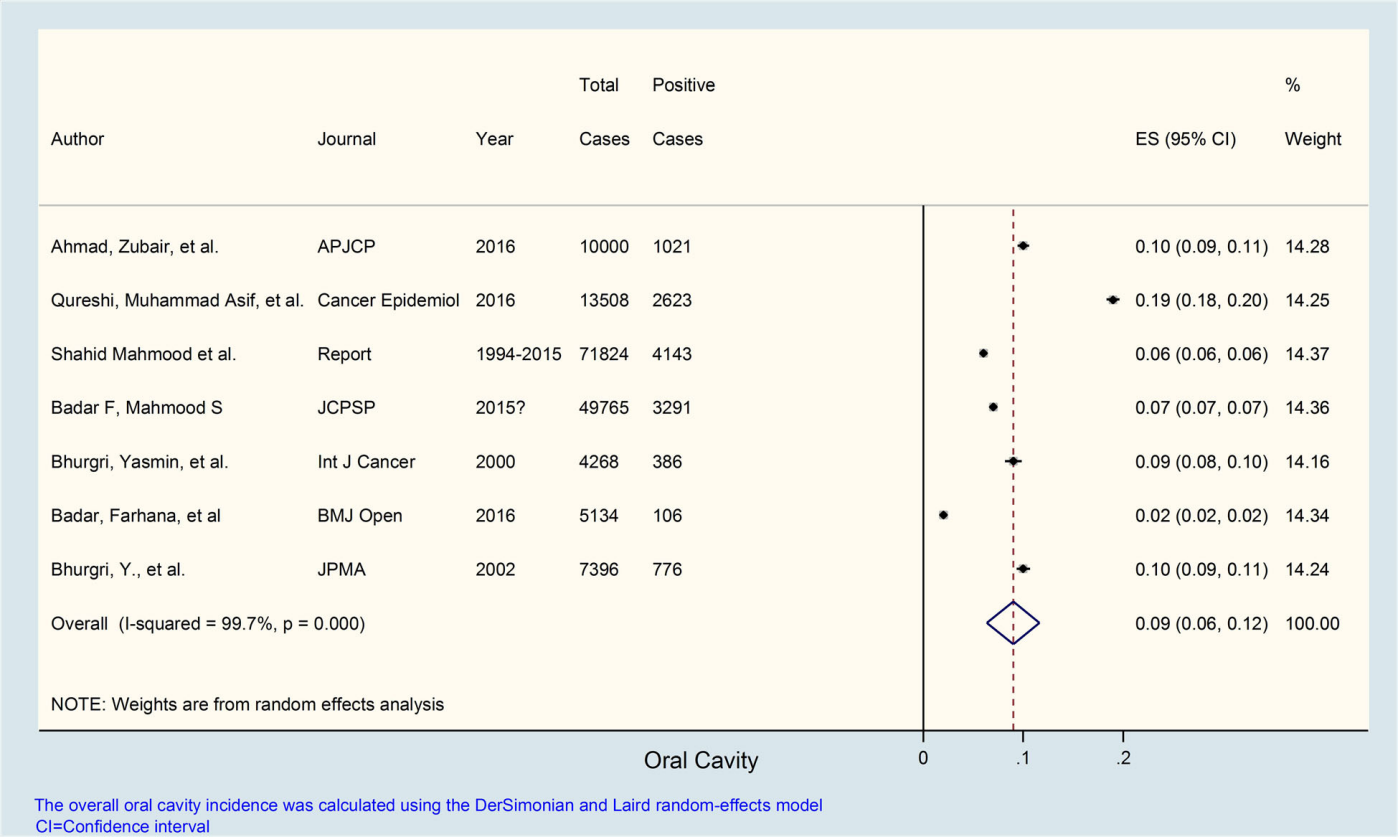
Prevalence of oral cancer in Pakistan. Forest plot showing prevalence (%) estimates with 95% confidence intervals and weights allocated to each study based on sample size. The overall pooled prevalence estimate was 0.09 or 9%; *I*^2^ = 99.7%: high heterogeneity; *p* value was significant (*p* = < 0.001)

Seven studies were evaluated for determining the prevalence of oral cancer. The prevalence of oral cancer in these studies which all carried statistical weights hovering around 14% ranged from 2 to 19% with an overall prevalence of 9%; *I*^2^ value was 99.7% indicating extremely high heterogeneity; and *p* value was < 0.001 which was markedly statistically significant (Fig. 1).

**Fig. 2:**
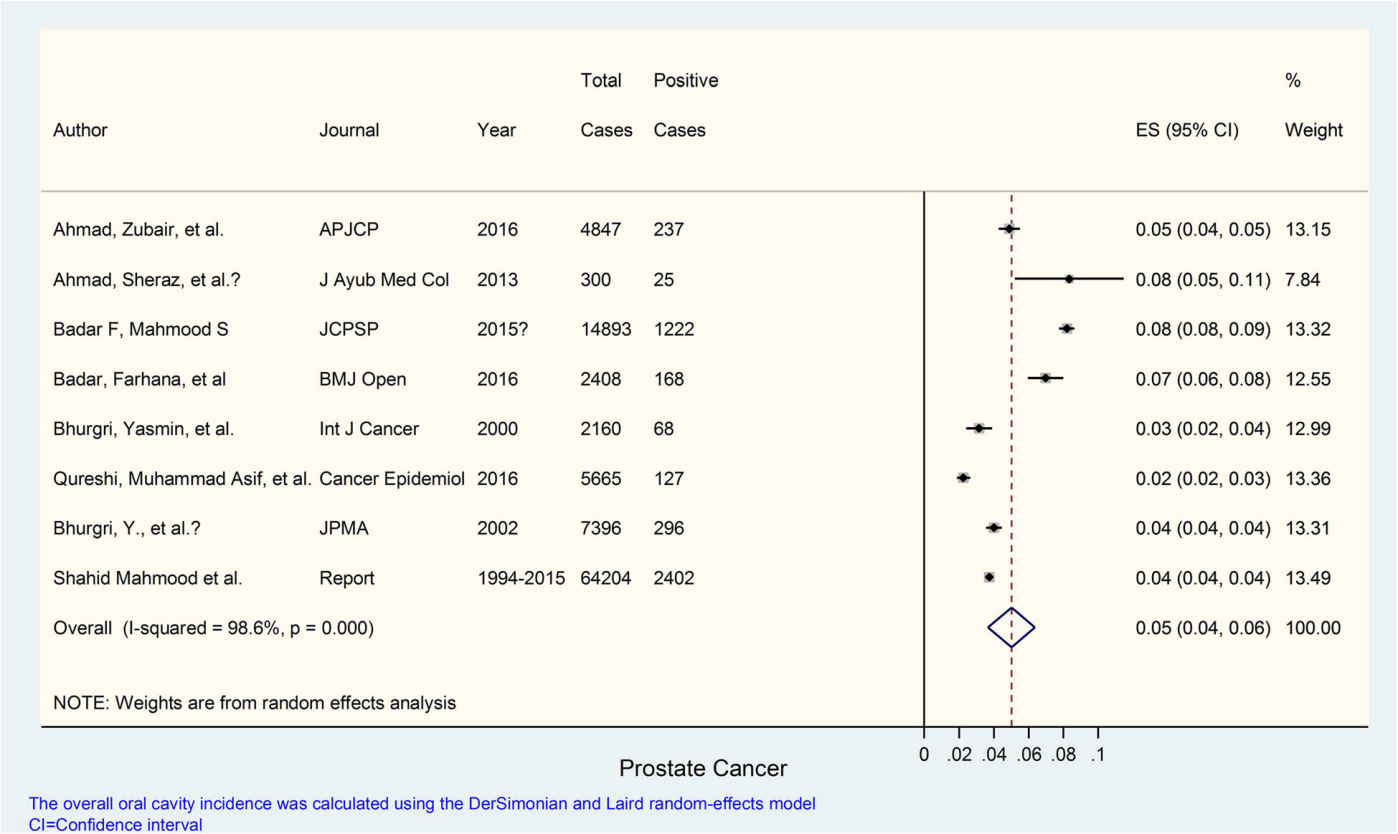
Prevalence of prostate cancer in Pakistan. Forest plot showing prevalence (%) estimates with 95% confidence intervals and weights allocated to each study based on sample size. The overall pooled prevalence estimate was 0.05 or 5%; *I*^2^= 98.6%: high heterogeneity; *p* value was significant (*p* = < 0.001)

Eight studies were evaluated for determining the prevalence of prostate cancer. The prevalence of prostate cancer in these studies most of which carried weights (according to their sample size) hovering around 13% (although one study carried a weight around 8%) ranged from 2 to 8% with an overall prevalence of 5%; *I*^2^ value was 98.6% indicating extremely high heterogeneity; and *p* value was < 0.001 which was markedly statistically significant (Fig. 2).

**Fig. 3:**
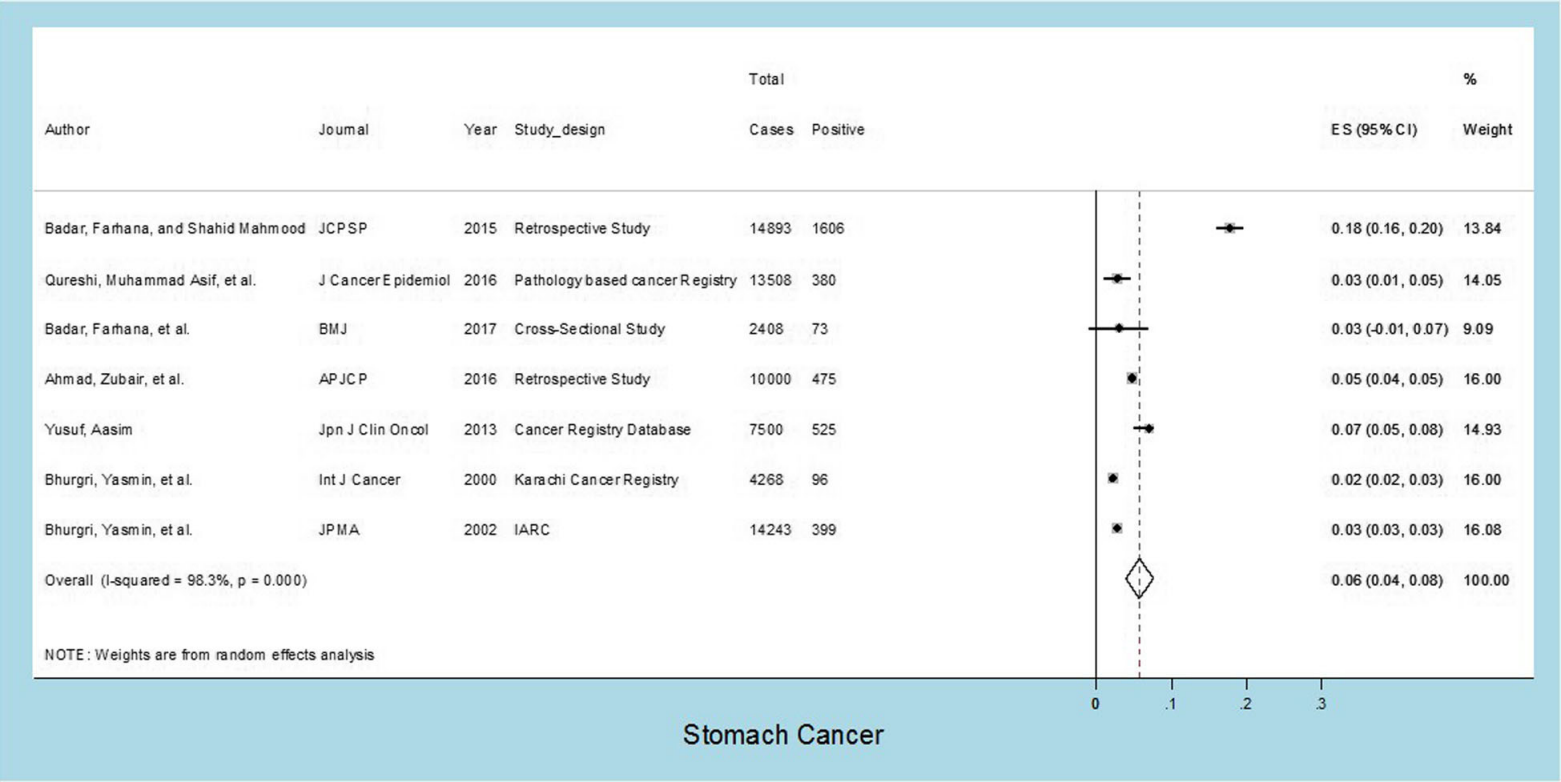
Prevalence of gastric cancer in Pakistan. Forest plot showing prevalence (%) estimates with 95% confidence intervals and weights allocated to each study based on sample size. The overall pooled prevalence estimate was 0.06 or 6%; *I*^2^= 98.3%: high heterogeneity; *p* value was statistically significant (*p* = < 0.001)

Seven studies were evaluated for determining the prevalence of gastric cancer. The prevalence of gastric cancer in these studies which all carried almost identical weights according to their sample size hovering around 15% ranged from 2 to 18% with an overall prevalence of 6%; *I*^2^ value was 98.3% indicating extremely high heterogeneity; and *p* value was < 0.001 which was highly statistically significant (Fig. 3).

**Fig. 4:**
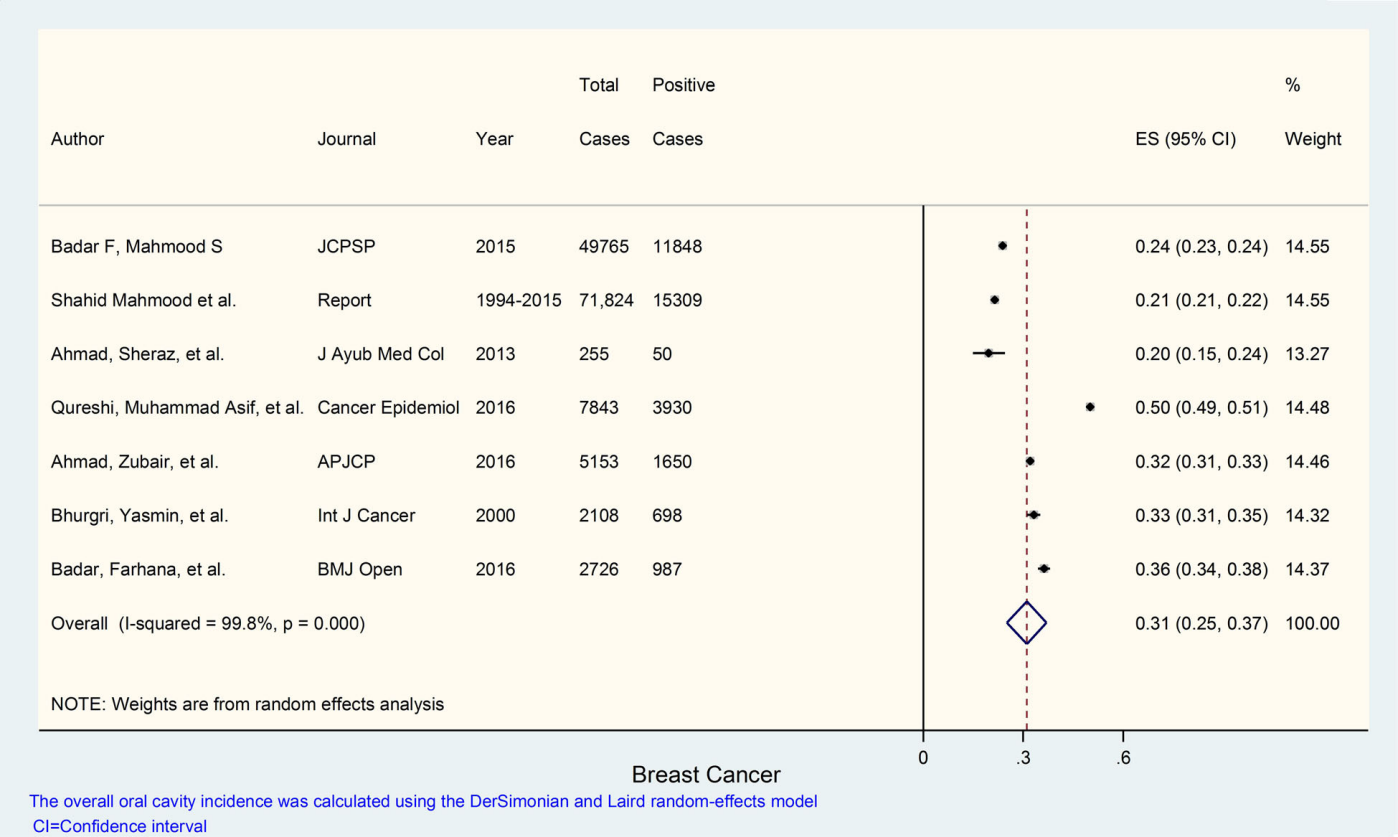
Prevalence of breast cancer in Pakistan. Forest plot showing prevalence (%) estimates with 95% confidence intervals and weights allocated to each study based on sample size. The overall pooled prevalence estimate was 0.31 or 31%; *I*^2^= 99.8%: high heterogeneity; *p* value was statistically significant (*p* = < 0.001)

Seven studies were evaluated for determining the prevalence of breast cancer. The prevalence of breast cancer in these studies most of which carried identical weights hovering around 14% according to their sample size ranged from 20 to 50% with an overall prevalence of 31%; *I*^2^ value was 99.8% indicating extremely high heterogeneity; and *p* value was < 0.001 which was highly statistically significant (Fig. 4).

**Fig. 5:**
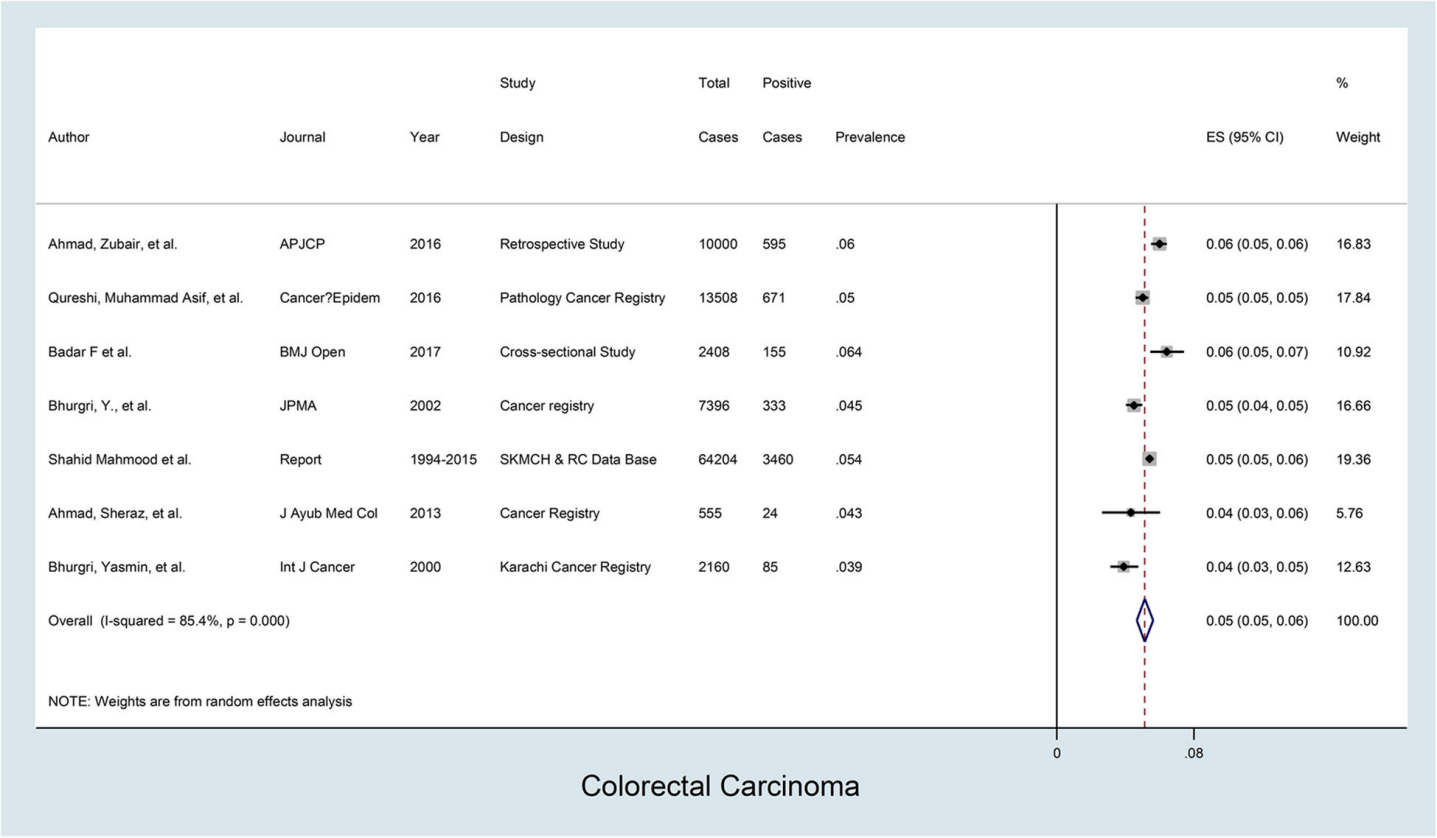
Prevalence of colorectal cancer in Pakistan. Forest plot showing prevalence (%) estimates with 95% confidence intervals and weights allocated to each study based on sample size. The overall pooled prevalence estimate was 0.05 or 5%; *I*^2^= 85.4%: high heterogeneity; *p* value was statistically significant (*p* = < 0.001)

Seven studies were evaluated for determining the prevalence of colorectal cancer. The prevalence of colorectal cancer in these studies which carried weights ranging from 5 to 19% according to their sample age, ranged from 4 to 6% with an overall prevalence of 5%; *I*^2^ value was 85.4% indicating very high heterogeneity; and *p* value was < 0.001 which was markedly statistically significant (Fig. 5).

The variation in data on commonest cancers obtained from different studies conducted in specific regions of the country is shown in Table 1.

**Table 1:** Variation in data on the commonest cancers obtained from different studies conducted in specific areas of Pakistan. (*n* = 13)

No.	Study name	Place of origin	Year/s for which cancer incidence determined	Year in which study was published	Total number of cases in study	Commonest cancers in males	Commonest cancers in females
1	Badar et al. (Punjab Cancer Registry)	Lahore, Central Punjab	2010–2012	2016	15,840	- Prostate - Urinary bladder - Trachea, bronchus, lung - NHL - CNS	- Breast - Ovary - Corpus uteri - NHL - Cervix
2	Qureshi et al. (Dow Diagnostic Research and Reference Laboratory, Dow University of Health Sciences)	Karachi, Sindh	2010–2015	2016	13,508	- Oral cavity and lip - Non-melanoma skin cancer - Colorectum - Esophagus - Larynx	- Breast - Oral cavity and lip - Esophagus - Colorectum - Non-melanoma skin cancer
3	Ahmad et al. (Aga Khan University Hospital)	Karachi, Sindh	2014	2016	10,000	- Oral cavity - NHL - Colorectum - Stomach - Esophagus	- Breast - Esophagus - NHL - Oral cavity - Ovary
4	Masood et al. (Pakistan Atomic Energy Commission Cancer Registry)	Lahore, Central Punjab	1984–2014	2015	80,390	- Head and neck - Central nervous system (CNS) - Non-Hodgkin lymphoma (NHL)	- Breast - Ovary - Cervix
5	Badar and Mahmood (Shaukat Khanum Memorial Cancer Hospital and Research Centre)	Lahore, Central Punjab	1994–2012	2015	55,974	- Liver and intrahepatic bile ducts - Lip and oral cavity - NHL	- Breast - Ovary - Lip and oral cavity
6	Ahmad S et al. (Ayub Medical College)	Abbottabad, Khyber Pukhtunkhwa	2007–2012	2013	555	- Hodgkin lymphoma - Leukemia - Prostate - Lung	- Leukemia - Breast - Lymphoma - Ovary - Stomach
7	Hanif et al. (Allama Iqbal Medical College)	Lahore, Central Punjab	1997–2001	2009	1500	- Leukemia - NHL - Lung	- Breast - Ovary
8	Atique et al. (Combined Military Hospital)	Multan, South Punjab	2002–2007	2008	930	- Leukemia - Prostate - Urinary bladder	- Leukemia - Breast - Skin
9	Bhurgri et al. (Aga Khan University Hospital; Zainab Punjwani Hospital and Jinnah Postgraduate Medical Center)	Larkana, Sindh	2000–2002	2006		- NHL - Oral cavity - Prostate - Liver - Urinary bladder	- Breast - Oral cavity - NHL - Skin - Thyroid
10	Bhurgri et al. (Aga Khan University Hospital)	Hyderabad, Sindh	1998–2002	2005		- Oral cavity - NHL - Lung - Urinary bladder - Prostate	- Breast - Oral cavity - Gall bladder - Esophagus - Cervix
11	Bhurgri et al. (Aga Khan University Hospital)	Karachi, Sindh	1998–1999	2002	11,368	- Oral cavity - Lung - NHL	- Breast - Oral cavity - Ovary
12	Bhurgri et al. (Sindh Medical College, Dow Medical College and Civil Hospital, JPMC, AKUH, Liaquat National Hospital, Zainab Punjwani Hospital, National Institute of Child Health, Baqai Institute of Oncology, Lady Dufferin Hospital, College of Physicians and Surgeons Pakistan)	Karachi, Sindh	1998–1999	2002	14,243	- Lung - Oral cavity - Larynx - Prostate - Urinary bladder	- Breast - Oral cavity - Gall bladder - Cervix - Ovary
13	Bhurgri et al. (Karachi Cancer Registry)	Karachi, Sindh	1995–1997	2000	4268	- Lung - Oral cavity - Larynx - Urinary bladder - NHL	- Breast - Oral cavity - Ovary - Cervix - Esophagus

The details of all the studies analyzed for determining the prevalence of the five cancer types are shown in Table 2.

**Table 2:** Details of studies analyzed for determining prevalence of five cancer types

								Prevalence with 95% confidence interval	
	Author	Journal	Year	Study design	Total cases	Positive cases	Overall	Lower	Upper
Breast Cancer	Badar F, Mahmood S	JCPSP	2015	SKMCH and RC database	49,765	11,848	24%	23.4%	24.2%
	Shahid Mahmood et al.	SKMCH Report	1994–2015	SKMCH and RC database	71,824	15,309	21%	21.0%	21.6%
	Ahmad, Sheraz, et al.	J Ayub Med Col	2013	Cancer registry	255	50	20%	14.7%	24.5%
	Qureshi, Muhammad Asif, et al.	Cancer Epidemiol	2016	Pathology cancer registry	7843	3930	50%	49.0%	51.2%
	Ahmad, Zubair, et al.	APJCP	2016	Retrospective study	5153	1650	32%	30.7%	33.3%
	Bhurgri, Yasmin, et al.	Int J Cancer	2000	Karachi Cancer Registry	2108	698	33%	31.1%	35.1%
	Badar, Farhana, et al.	BMJ Open	2016	Cross-sectional study	2726	987	36%	34.4%	38.0%
Oral Cancer	Ahmad, Zubair, et al.	APJCP	2016	Retrospective study	10,000	1021	10.0%	9.4%	10.6%
	Qureshi, Muhammad Asif, et al.	Cancer Epidemiol	2016	Pathology cancer registry	13,508	2623	19.0%	18.3%	19.7%
	Shahid Mahmood et al.	Report	1994–2015	SKMCH and RC database	71,824	4143	6.0%	5.8%	6.2%
	Badar F, Mahmood S	JCPSP	2015	SKMCH and RC database	49,765	3291	7.0%	6.8%	7.2%
	Bhurgri, Yasmin, et al.	Int J Cancer	2000	Karachi Cancer Registry	4268	386	9.0%	8.1%	9.9%
	Badar, Farhana, et al.	BMJ Open	2016	Cross-sectional study	5134	106	2.0%	1.6%	2.4%
	Bhurgri, Y., et al.	JPMA	2002	Cancer registry	7396	776	10.0%	9.3%	10.7%
Prostate Cancer	Ahmad, Zubair, et al.	APJCP	2016	Retrospective study	4847	237	4.9%	4.3%	5.5%
	Ahmad, Sheraz, et al.	J Ayub Med Col	2013	Cancer registry	300	25	8.3%	5.2%	11.5%
	Badar F, Mahmood S	JCPSP	2015	SKMCH and RC database	14,893	1222	8.2%	7.8%	8.6%
	Badar, Farhana, et al.	BMJ Open	2016	Cross-sectional study	2408	168	7.0%	6.0%	8.0%
	Bhurgri, Yasmin, et al.	Int J Cancer	2000	Karachi Cancer Registry	2160	68	3.1%	2.4%	3.9%
	Qureshi, Muhammad Asif, et al.	Cancer Epidemiol	2016	Pathology cancer registry	5665	127	2.2%	1.9%	2.6%
	Bhurgri, Y., et al.	JPMA	2002	Cancer registry	7396	296	4.0%	3.6%	4.4%
	Shahid Mahmood et al.	Report	1994–2015	SKMCH and RC database	64,204	2402	3.7%	3.6%	3.9%
Stomach Cancer	Qureshi, Muhammad Asif, et al.	Cancer Epidemiol	2016	Pathology cancer registry	13,508	380	2.8%	2.5%	3.1%
	Ahmad, Zubair, et al.	APJCP	2016	Retrospective study	10,000	475	5%	4%	5%
	Bhurgri, Yasmin, et al.	Int J Cancer	2000	Karachi Cancer Registry	4268	96	2.2%	1.8%	2.6%
	Badar, Farhana, et al.	BMJ Open	2016	Cross-sectional study	5134	73	1.4%	1.1%	1.7%
	Bhurgri, Y., et al.	JPMA	2002	Karachi Cancer Registry	7396	203	2.7%	2.3%	3.1%
	Yusuf	Jpn J Clin Oncol	2013	SKMCH Cancer Registry Database	7500	525	7%	5%	9%
	Badar et al.	JCPSP	2015	Retrospective study	14,893	1606	18%	16%	20%
Colorectal Cancer	Qureshi Muhammad Asif, et al.	Cancer Epidemiol	2016	Pathology cancer registry	7843	671	5%	4.6%	5.5%
	Bhurgri Yasmin, et al.	Int J Cancer	2000	Karachi Cancer Registry	2108	150	7.1%	6.8%	7.5%
	Ahmad Zubair, et al.	APJCP	2016	Retrospective study	5153	595	5.9%	5.5%	6.4%
	Badar F, et al.	BMJ Open	2017	Cross-sectional Study	2408	155	6.4%	6.0%	6.9%
	Ahmad Sheraz, et al.	JAMC	2013	Cancer registry	555	24	4.3%	4.0%	4.8%
	Shahid Mahmood, et al.	Report	1994–2015	SKMCH+RC Database	64,204	3460	5.4%	5.0%	5.8%
	Bhurgri Yasmin, et al.	JPMA	2002	Cancer registry	7396	333	4.5%	4.0%	5.0%

## Discussion

The last census in Pakistan was carried out in 1998 [16]. A countrywide census is being currently conducted after a gap of 19 years. However, its findings are yet to be made public. Pakistan’s population was around 173 million in 2008 and is now estimated to be greater than 190 million. Of the six large ethnic groups which constitute the bulk of the population, Punjabis are by far the largest and mainly inhabit the central part of the country extending towards the east, lower north, and upper south. The Sindhis mainly inhabit the south and southwest while the Muhajirs (people who migrated from the Muslim majority provinces of India at the time of partition of India in 1947) are mainly concentrated in the lower south. The Baloch are mostly concentrated in the southwest while the Pashtuns mainly inhabit the north. However, a significant proportion of Pashtuns are also concentrated in the southwest. Similarly, significant percentages of Punjabis also inhabit the south and southeast. In addition, there are several relatively minor groups such as the Kashmiris and the Baltits in the upper north. Since all these ethnic groups are very diverse and heterogeneous, cancer figures obtained from different regions of the country (populated by particular ethnic groups) may vary considerably. There is no national cancer registry and only two regional cancer registries. One of these, the Punjab Cancer Registry [5] is Lahore based. It was established in 2005 and mainly covers the ethnic Punjabi population of the city of Lahore and its surrounding areas. Lahore is Pakistan’s second largest city and is located in the Punjab heartland in the central part of the country. This registry was awarded Associate Mentor status by the International Association of Cancer Registries in 2011. The other regional cancer registry, the Karachi South Cancer Registry [6], is based in Karachi, Pakistan’s largest city with a population of over 20 million. This registry was established in 1996. Karachi is Pakistan’s only truly metropolitan city, and although the most dominant ethnic group constituting the population of the city are the Muhajirs, people from all over Pakistan have migrated to and settled in Karachi in order to earn their livelihood. Thus, other ethnic groups constitute a significant proportion of the city’s population and Karachi, Pakistan’s commercial and financial hub, is often known as ‘mini Pakistan.’ Of Karachi’s five districts, two have a predominantly Muhajir population while the other three are more ethnically diverse. The District South is probably the most ethnically diverse district and cancer figures from Karachi South Cancer Registry may provide a more accurate picture regarding the incidence and prevalence of different cancers in Pakistan compared to the other studies. However, in the absence of a national cancer registry, nothing can be said with any degree of certainty. Accurate population-based data on cancer incidence and mortality is essential for sensible allocation of precious health care resources. However, in present day Pakistan, with its burgeoning and uncontrolled population growth, economic meltdown and political uncertainty, natural calamities such as earthquakes and floods resulting from climate change, poverty, and rampant infectious diseases, very scarce resources allocation to health (and education) with bulk of resources diverted to the military due to security concerns means that the setting up of a national population-based cancer registry and obtaining valid cancer statistics from such a registry appears unlikely in the near future. However, major pathology centers in the country (including ours) are now pooling our resources and coordinating our efforts to devise a system which can record accurate data on cancer incidence and mortality, and ensure that no new cancer case is missed while minimizing duplication and over-reporting of the cancer burden. We hope that this effort by major pathology centers will be successful as pathology-based cancer data can be very useful in determining patterns of cancer within a population provided that information of patient demographics is accurate.

Globocan 2008 [11] and 2012 [12] roughly estimated the incidence, prevalence, and mortality rates of different cancers in Pakistan by computing gender, site, and age-specific incidence rates as population weighted averages of the served rates in South Karachi (1998–2002), Punjab, Lahore district (2008–2010), and Quetta (1998–1999) incidence rates, the estimate for India and the national estimate for Iran (2008). The weighted averages represent the urban and rural populations of Sindh, Punjab, and Baluchistan provinces. The incidence rates were corrected for 40% under reporting. From these estimated urban gender, age, and site-specific incidence rates, the rural cancer incidence rates were derived using estimated urban: rural ratios of age-standardized incidence rates (ASRS) in India in 2012. Similarly, the prevalence and mortality were also roughly computed in a roundabout way by comparing the incidence estimates with the regional average of observed survival in different cancers and age groups. Based on these, Globocan estimated that the number of new cancers was 159,577.

We feel that due to the marked ethnic heterogeneity of the Pakistani population and the absence of a national cancer registry, the data on the incidence and prevalence of various cancers in Pakistan is very variable depending on the region where a particular study was carried out and the predominant population of that particular region. Variations in the cancer data obtained from various studies conducted in different parts of the country are shown in Table 1 and appear to be quite marked and significant. The data for analyzing the prevalence and heterogeneity of specific types of cancer was obtained from 13 studies of which 5 were from Karachi [1, 6, 7, 15], 4 from Lahore [4, 5, 14], and 1 each from the Hazara Division in Khyber Pakhtunkhwa, Multan Division in Punjab, Larkana Division in Sindh, and Hyderabad Division in Sindh, respectively [2, 3, 9, 10].

The prevalence of specific cancer types in different studies is summarized in Table 2 while details are provided in Figs. 1, 2, 3, 4, and 5. These show that prevalence rates of different common cancer types show marked variations in different studies depending on the place of origin of the study and that these variations are statistically significant. For example, the prevalence of breast cancer in females ranged from 20 to 50% in various studies with overall pooled prevalence of 31% and extremely high heterogeneity with *I*^2^ value of 99.8%. Similarly, the prevalence of oral cavity (and lip) cancer ranged from 2 to 19% with overall pooled prevalence of 9% and extremely high heterogeneity with *I*^2^ value of 99.7%. The prevalence of prostate cancer in males ranged from 2 to 8% with overall pooled prevalence of 5% and extremely high heterogeneity with *I*^2^ value of 98.6%. The prevalence of gastric cancer ranged from 2 to 18% in various studies with overall pooled prevalence of 6% and extremely high heterogeneity with *I*^2^ value of 98.3%. Finally, the prevalence of colorectal cancer in both males and females ranged from approximately 4 to 6% in various studies with overall pooled prevalence of approximately 5% and very high heterogeneity with *I*^2^ value of 85.4%.

The significant differences in the frequency of various cancer types seen in the different studies reflect the heterogeneity in the ethnic makeup of the population in the different regions. We believe that most of the heterogeneity is genetic in origin and is primarily and largely based on the presence of significant genetic differences among the various subsets of Pakistan’s population. However, environmental factors also contribute to the development of specific cancers in some populations. Incidence of oral cancer in various studies varied from as low as 2% to as high as 19% (Table 2). At least in oral cancer, environmental factors (mainly ingestion or inhalation of various carcinogenic substances routinely by men and women, old and young, of various ethnic groups) play an important and definitive role in making oral cancer one of the commonest cancers in most areas of Pakistan. Large percentages of Urdu speaking Muhajir (36% males and 44% females according to one study) settled in Karachi and Hyderabad chew paan (betel leaves and betel nuts along with lime) or paan with tobacco and males smoke not just cigarettes but also hand-rolled cigarettes called bidis. Both males and females especially Sindhis in rural areas of Sindh (Southern Pakistan) smoke hookas (tobacco inhaled through a tube immersed in water) while pathan males from Northern Pakistan (including those who are settled in Karachi) take tobacco in the form of a coarse powder (gutka and niswar) or inhale it in the form of snuff powder. Children in Karachi belonging to all ethnic types ingest tobacco mixed with lime and flavoring agents (mawa, zarda, etc.). All these are major risk factors for oral cancers, and it reflects in the high incidence of oral cancers in Karachi and Sindh. However, levels in Sindh are lower compared to Karachi owing to much less chewing of paan among ethnic Sindhis compared to Muhajirs. Similarly, oral cancer is very common in Northern and Southwestern Pakistan (where Pathans are settled) due to their habit of taking niswar as noted above. The southwest part of Pakistan (in Balochistan province) lies on the Asian esophageal cancer belt which also comprises of Iran, Turkey, Iraq, Mongolia, and China. We receive the largest percentage of esophageal cancers in our practice from Quetta, the capital of Baluchistan while incidence of esophageal cancer in Punjab and other parts of Pakistan is low. This indicates the presence of geographic and ethnic factors in the pathogenesis of esophageal cancer in Pakistan. Consumption of scalding hot tea and other beverages and hot food in these areas also contribute. Incidence of gastric cancer in various studies varied from as low as 1.4% to as high as 18% (Table 2). We believe that both genetic differences in the study populations as well as environmental (such as dietary) factors in different areas play a major role in causing these variations. Gastric cancer is prevalent in most ethnic groups in Pakistan most likely due to high incidence of chronic helicobacter infections (owing to overcrowding, lack of sanitation, poor hygiene, etc.) across the ethnic divide. However, incidence is not as high as it should have been indicating a role of dietary protective factors. Incidence of colorectal cancer in various studies from different areas of Pakistan ranged from 4 to 6.8%. Again, we believe that genetic difference play a part in causing these variations. However, environmental factors especially dietary factors may play a major role. High incidence of colorectal cancer in Pakistan along multiethnic lines can be explained by dietary factors such as consumption of large quantities of red meat especially beef as well as fat. Specifically, the incidence of colorectal cancer is very high in Northern and Southwestern Pakistan due to the consumption of smoked meat by the Pathans and Balochis in these areas. However, incidence rates of colorectal cancer are lower in Karachi where majority of the population cannot afford to eat meat regularly. Incidence of breast cancer in various studies from different areas of Pakistan ranged from 20% to as high as 50% (Table 1), indicating what we believe to be mainly genetic differences in the study populations. Although breast cancer is extremely common in Pakistani females, the genetic factors associated with high incidence of breast cancer in various ethnic groups remain unexplored. The reproductive factors implicated in the development of breast cancer may not apply to Pakistani females as majority have high fertility (more than three births); puberty usually occurs at a young age, and majority of females especially in rural areas breast feed their babies for a considerable time. However, lately with the lifestyle becoming more urban and westernized, factors such as lack of breast feeding, nulliparity, late age at pregnancy, prolonged and excessive use of oral contraceptives, and obesity may be contributing to the increase in breast cancer cases in the country. Cervix cancer levels are low in most studies from Pakistan as religious and social issues prevent exposure to established risk factors such as multiple sex partners. Incidence of prostate cancer in various studies from different areas of the country with marked ethnic heterogeneity ranged from as low as 2.2% to as high as 8.8% (Table 2). Again, genetic factors probably account for these variations. It is not known what protective factors in Pakistani males contribute to relatively low incidence of prostate cancer in Pakistan. [1, 2, 4–6, 8, 15, 16].

## Conclusion

The findings in this study demonstrate that the prevalence rates of different cancer types show marked variation in different studies depending on the place of origin of the study and dominant ethnic group in the region, and these variations are highly statistically significant. Our findings closely reflect the marked heterogeneity of the country’s population. The *I*^2^ and *p* values for individual cancers reported from different areas with differences in predominant ethnic population groups demonstrate extremely high heterogeneity and are statistically significant. Additional studies from different regions of Pakistan need to be collected and analyzed to get a clearer picture of variation in cancer incidence and prevalence in Pakistan owing to the marked heterogeneity of ethnic types in different regions of the country. A national cancer registry needs to be established urgently.

## Abbreviations

ASRS: Age-standardized incidence rates
MOOSE: Meta-analysis of Observational Studies in Epidemiology
